# NeuroHeal Improves Muscle Regeneration after Injury

**DOI:** 10.3390/cells10010022

**Published:** 2020-12-24

**Authors:** Sara Marmolejo-Martínez-Artesero, David Romeo-Guitart, Vanesa Venegas, Mario Marotta, Caty Casas

**Affiliations:** 1 Department of Cell Biology, Physiology and Immunology, Institut de Neurociències (INc), Universitat Autònoma de Barcelona (UAB), Bellaterra, 08193 Barcelona, Spain; Sara.Marmolejo@uab.cat; 2 Laboratory “Hormonal regulation of brain development and functions”—Team 8, Institut Necker Enfants-Malades (INEM), INSERM U1151, Université Paris Descartes, Sorbonne Paris Cité, 75015 Paris, France; 3 Leitat Technological Center, Carrer de la Innovació 2, Terrassa, 08225 Barcelona, Spain; vanesa.venegas@vhir.org (V.V.); mmarotta@leitat.org (M.M.); 4 Bioengineering, Cell Therapy and Surgery in Congenital Malformations Laboratory, Vall d’Hebron Institut de Recerca (VHIR), Universitat Autònoma de Barcelona (UAB), 08035 Barcelona, Spain

**Keywords:** muscle regeneration, NeuroHeal, satellite cells, Sirtuin 1, sport injury

## Abstract

Musculoskeletal injuries represent a challenging medical problem. Although the skeletal muscle is able to regenerate and recover after injury, the process engaged with conservative therapy can be inefficient, leading to a high re-injury rate. In addition, the formation of scar tissue implies an alteration of mechanical properties in muscle. There is still a need for new treatments of the injured muscle. NeuroHeal may be one option. Published studies demonstrated that it reduces muscle atrophy due to denervation and disuse. The main objective of the present work was to assess the potential of NeuroHeal to improve muscle regeneration after traumatic injury. Secondary objectives included characterizing the effect of NeuroHeal treatment on satellite cell biology. We used a rat model of sport-induced injury in the gastrocnemius and analyzed the effects of NeuroHeal on functional recovery by means of electrophysiology and tetanic force analysis. These studies were accompanied by immunohistochemistry of the injured muscle to analyze fibrosis, satellite cell state, and fiber type. In addition, we used an in vitro model to determine the effect of NeuroHeal on myoblast biology and partially decipher its mechanism of action. The results showed that NeuroHeal treatment advanced muscle fiber recovery after injury in a preclinical model of muscle injury, and significantly reduced the formation of scar tissue. In vitro, we observed that NeuroHeal accelerated the formation of myotubes. The results pave the way for novel therapeutic avenues for muscle/tendinous disorders.

## 1. Introduction

Musculoskeletal injuries represent a challenging problem, being the most common cause of severe long-term injuries, accounting for 10 to 55% of all sports-related injuries [1]. Muscle injuries are classified by cause of trauma and grade: an acute lesion caused by a direct trauma, such as muscle lacerations, an indirect trauma, such as strains, or by chronic disease or degenerative diseases, such as muscular dystrophies [2]. To repair this injury, skeletal muscle has regenerative capabilities to self-repair after trauma or in response to muscular dystrophies. This muscle reparative mechanism is divided into different phases: degeneration, inflammation, regeneration, remodeling, and innervation [3]. The regenerative process is mainly mediated by a specific type of stem cell, the satellite cell (SC) [4,5], which, after being activated, follows a process of differentiation giving a newly formed or recovered myofiber [6]. Recent reports have provided evidence that SCs are essential for the regenerative process, which is directly dependent on the microenvironmental niche [7,8].

Current therapies for most muscle injuries are mainly directed to decrease pain and inflammation by NSAIDs drugs, supplemented with immobilization and external physical stimulation. In severe cases, surgery is required to restore the anatomic continuity and function. In some cases, the endogenous process of muscle repair proves insufficient, leading to loss of contractile tissue, fatty degeneration, and fibrotic scar tissue, which can cause long-term deficits in muscle structure and strength. Although in those cases medical need is unsolved, some promising therapies are under study. The use of molecular therapies, such as growth factors or platelet-rich-plasma (PRP), which releases growth factors, structural proteins, and interleukins and chemokines, try to foster a beneficial microenvironment to boost regenerative biological processes [3]. Although there is controversy regarding the clinical effects of PRP in humans [9], it was shown to promote beneficial effects on muscle recovery in a rat injury model [10]. Moreover, cell therapy is a promising approach to treat skeletal muscle injuries in pre-clinical settings [11], but not for all types of injuries. Therefore, all current available pharmacological and biological agents only relieve clinical symptoms and have limited or no effect on the progression of the underlining muscle disease.

In this study, we evaluated the therapeutic potential of NeuroHeal, a combination of two repurposed drugs (Acamprosate plus Ribavirin), previously designed using artificial intelligence [12,13]. Drug repurposing for other indications facilitates its readiness for clinical use, since their PD and PK are well-known in humans. We have previously reported its neuroprotective effects in different neurodegenerative models [12,14,15,16]. In addition, we recently observed that NeuroHeal protects the muscle by reducing atrophy induced by denervation or by immobilization [17], suggesting it may have also protective effects in other types of myopathies. NeuroHeal neuroprotective and neurodegenerative effects involve SIRT1 activation, and autophagy modulation [14,15,18]. Moreover, we have recently described that boosting SIRT1 activity is essential in the mechanism of action of NeuroHeal to reduce muscle atrophy [17]. Herein, we aimed to elucidate whether NeuroHeal has an effect on muscle regeneration by using an in vivo model of surgically-induced lesion which mimics the most frequent skeletal muscle lesions observed in human sports clinics. We also provide insight into the possible mechanism involved in the pro-regenerative effect of NeuroHeal.

## 2. Materials and Methods

### 2.1. Muscle Injury Model

All the experimental procedures were performed in accordance with Spanish (Real Decreto 53/2013) and European (2010/63/UE) legislation, approved by the Departament d’Agricultura, Ramaderia, Pesca, Alimentació i Medi Natural of the Catalan Government (Generalitat de Catalunya) and followed the ethical standards in sport and exercise science research [10,19,20]. The work was performed under the protocol number 2017/52.17 approved by the Institutional Animal Care and Use Committee at Vall d’Hebron Hospital Research Institute (Barcelona, Spain). Adult male Wistar rats 8 weeks of age (Harlan Laboratories) were used for the in vivo studies. Rats were anesthetized by an intraperitoneal (i.p.) injection of a mixture of ketamine (90 mg/kg, Ketaset) and xylazine (10 mg/kg, Rompun). The surgical procedure was performed as previously described [10,20], inserting an 18 G biopsy needle in the right medial gastrocnemius (GA) muscle at 3 mm from the muscle-tendon junction. Post-surgical analgesia (0.01 mg/kg buprenorphine) was administered to all operated animals for 24 h after surgically-induced skeletal muscle injury.

### 2.2. Drug Treatment

NeuroHeal mixture is composed of Acamprosate and Ribavirin. For in vivo experiments, we grounded Acamprosate (Merck, Darmstadt, Germany) and Ribavirin (Normon, Madrid, Spain) pills into a fine powder and daily administered 40 mg/kg and 26 mg/kg, respectively, by gavage, dissolved in 1 mL of water at a concentration of 2.2 mM Acamprosate and 1 mM Ribavirin. To test whether NeuroHeal may accelerate muscle regeneration after damage, we used a model of injured GA muscle, comparing a group of rats treated with NeuroHeal (*n* = 5) and a group of rats receiving vehicle (*n* = 5), by gavage administration.

### 2.3. Electrophysiology

Rats were anesthetized by i.p. administration of ketamine and xylazine, and kept warm with a thermostated blanket. The sciatic nerve was stimulated with single pulses of increasing intensity by means of transcutaneous electrodes placed at the sciatic notch. The compound muscle action potential (CMAP) of the GA muscle was recorded to measure the maximum CMAP amplitude. The tests were performed using an electromyography (EMG) apparatus (Synergy Medelec, Viasys HealthCare). The CMAP amplitude from baseline to peak was used as a readout of the GA muscle integrity. The percentage of CMAP recovery was calculated as the ratio of injured/uninjured contralateral values. The nerve conduction test was performed before surgical injury and at 3, 7, and 14 days post-injury (dpi).

### 2.4. Measurement of Muscle Force

To measure contractile muscle force, rats were anesthetized by i.op. injection of ketamine and xylazine. Animals were placed in a prone position and the limbs were immobilized. The Achilles tendon was separated from the calcaneus and attached to a force transducer (Wide Range Force Transducer, MLT 1030/D; ADInstruments, Sydney, Australia) connected to a PowerLab/16SP data acquisition hardware (ADInstruments). The sciatic nerve was exposed by lateral incision and an electrode placed around the nerve and connected in turn to a stimulator (Stimulus Isolator, FE180; ADInstruments). The GA muscles were covered with mineral oil (Sigma-Aldrich, Saint Louis, MO, USA) to prevent drying, and the room temperature was set at 25 °C. Muscle force was measured for right (injured) and left (control) GA muscles of each rat. Repeated isometric muscle twitches were induced at a frequency of 1 Hz (5 pulses) and a voltage of 5 V. The twitch response was analyzed by contraction time (CT) and half-relaxation time (HRT). Following twitch stimulation or peak force (PF), maximum tetanus force (TetF) was induced by a train of stimuli with a frequency of 100 Hz, a pulse width of 0.1 ms and a voltage of 5 V. The percentage of the TetF was calculated with the percentage of the ipsi versus the contra GA of each animal. Animals were euthanized by anesthetic overdose immediately after finishing muscle force measurements and muscle samples were excised and processed for further histological analysis.

### 2.5. Histology

For histology studies, animals were euthanized by i.p. injection of an anesthetic overdose. The GA muscles were carefully excised, weighed, and immediately frozen in 2-methylbutane (Alfa Aesar, Haverhill, MA, USA), which was previously supercooled in liquid nitrogen, and were stored at −80 °C until further analysis.

To locate the area of injury, 10 µm serial cross sections were made from the myotendinous junction. Muscle samples were embedded in Tissue-tek, sectioned in a cryotome (Leica, Heidelberg, Germany), and preserved at −20 °C until analysis. Hematoxylin and Eosin (H&E) staining was performed in each series to cover 3 mm of the muscle to localize the injury. Each series was formed by five slices, having discarded 50 µm between them. The samples to be compared were processed together the same day within the same slide, and image analysis for all groups was also performed the same day using the same microscope setting.

For the H&E, nuclei were stained with Harris hematoxylin for 6 min followed by differentiation with an acid solution of 0.01% HCl in ethanol. The cytoplasm was stained with eosin for 1 min. Sections were dehydrated by graded ethanol (50%, 70%, 96%, and 100%, and xylene twice, 5 min in each solution) and mounted with DPX mounting solution.

For immunofluorescence labeling, the slices were pre-treated with frozen acetone, to fix the samples, and with 10 mg/mL NaBH_4_, to reduce autofluorescence for 80 min at 4 °C. After washing with standard Phosphate Buffered Saline (PBS), the tissue was incubated in blocking solution (0.3% Triton-X-100 and 10% fetal bovine serum in PBS) for 1 h at room temperature. Samples were then incubated at 4 °C with primary antibodies containing solution (PBS with 0.15% triton X-100, 5% FBS) overnight. The antibodies used were mouse anti-collagen type 1 (1:100, DBSH, Iowa City, IA, USA), mouse anti-developmental myosin heavy chain (dMyHC. 1:100, DSHB), mouse anti-fast myosin heavy chain (fMyHC. 1:100, DSHB), mouse anti-laminin (1:100, DSHB), mouse anti-MyoD (1:150; Santa Cruz, Dallas, TX, USA), mouse anti-Myogenin (1:100; Abcam, Cambridge, UK), rabbit anti-parvalbumin (1:100, Swant, Marly, Switzerland), mouse anti-Pax7 (1:100; DSHB), and mouse anti-αβ slow myosin heavy chain (sMyHC. 1:100, DSHB). After several washes with 0.3% Triton-X-100 in PBS, we added Alexa Fluor 488 and 594 conjugated secondary antibodies against the primary antibody (1:100; Jackson Immunoresearch, Cambridge, UK) and incubated for 1 h at room temperature. Counter-staining was performed with DAPI (Sigma) and mounted with Fluoromont (SouthernBiotech, Birmingham, UK) mounting solution. Images from different groups were taken under the same exposure time, sensibility, resolution, and microscope for each analyzed marker. Images were taken using a Nikon Eclipse Ni-E microscope equipped with a digital camera (Nikon DS-RiE, Nikon, Tokyo, Japan) and Nikon NIS-Element BR software (version 5.11.03, Nikon, Tokyo, Japan). Co-labeled fibers were determined as positive using a pseudocolor display by Image J software (version 1.46; National Institutes of Health), using images at 4× (fiber type markers) and 10× (myogenesis markers). For signal intensity analysis, randomly selected images were selected at 10× (collagen I) and 20× (parvalbumin), were transformed to grayscale, and after defining a threshold for background correction, immunoreactivity was analyzed by calculating the integrated density of a region of interest (ROI) and the total area of the image. For collagen I, the ROI was 1.49 mm^2^ and the ROI of parvalbumin was myofiber area for a total of 20 myofibers.

### 2.6. Myoblast Differentiation and Analysis

C2C12 myoblast cell line was grown in a medium composed of modified Eagle’s medium high-glucose (DMEM, Life Technologies, Carlsbad, CA, USA) supplemented with 10% fetal bovine serum (Sigma-Aldrich), and 1% penicillin/streptomycin solution (Sigma-Aldrich). Cells were kept in a humidified incubator at 37 °C under 5% CO_2_. To initiate the experiments, cells were seeded at a density of 8.5 × 10^3^ cell/mL, and after 24 h of culture and 80% of confluency, the medium was changed to a differentiation medium (DMEM supplemented with 2% horse serum (Sigma-Aldrich) with 1% penicillin/streptomycin solution), with or without NeuroHeal (55 μM acamprosate and 1 μM ribavirin) and Ex-527 (Sigma-Aldrich) at 10 μM, which was changed every 2 days, until the end of the experiment. At 0 (before changing to differentiation medium), 1, 3, and 5 div (days of in vitro) cells were fixed using 4% formaldehyde for 30 min and then washed out with TBS. For immunofluorescence labeling, cells were incubated in a blocking solution (0.3% Triton-X-100 and 10% normal donkey serum in TBS) for 1 h at room temperature. Samples were then incubated for 4 h at room temperature with primary antibodies containing solution (TBS with 0.3% triton X-100, 5% FBS). The antibodies used were rabbit anti-ki67 (1:100; Abcam), mouse anti-MyoD (1:150; Santa Cruz Biotechnology), mouse anti-Myosin heavy chain (all fast isoforms) (MyHC, 1:20, DSHB), rabbit anti-parvalbumin (1:100, Swant), and mouse anti-Pax7 (1:100; DSHB). After several washes in TBS-0.1% tween-20, we added Cy2 and Cy3-labeled secondary antibodies (1:200; Jackson Immunoresearch) were added and incubated for 50 min at room temperature. Counter-staining was performed with DAPI (Sigma) and mounting with Mowiol mounting medium (Southern Biotech). Images of C2C12 cells from different groups were taken at 20× under the same exposure time, sensibility, and resolution for each marker analyzed with the same microscope system as above. Co-labeled fibers were determined as positive using a pseudocolor display by Image J software. For the fusion coefficient, a mature myotube was considered having three or more MyHC positive nuclei. For signal intensity analysis, images were transformed to grayscale and analyzed immunoreactivity by calculating the integrated density of a ROI, after defining a threshold for background correction. For MyoD, the ROI used was DAPI to analyze nuclei area and for parvalbumin, the ROI used was 5615.103 µm^2^ for 15–20 myotubes for each condition.

### 2.7. Statistical Analysis

We performed an unpaired Student’s *t*-test to compare two groups, a one-way analysis of variance (ANOVA) to compare three or more groups, and a two-way ANOVA to compare grouped data followed by Tukey’s multiple comparison test, as appropriate. Data are presented as means ± standard error of the mean (SEM) and differences were assumed to be significant for *p* ≤ 0.05. Statistical analyses were conducted using GraphPad Prism 8 software.

## 3. Results

### 3.1. Text

#### 3.1.1. NeuroHeal Accelerates Muscle Fiber Function Recovery

**Figure 1:**
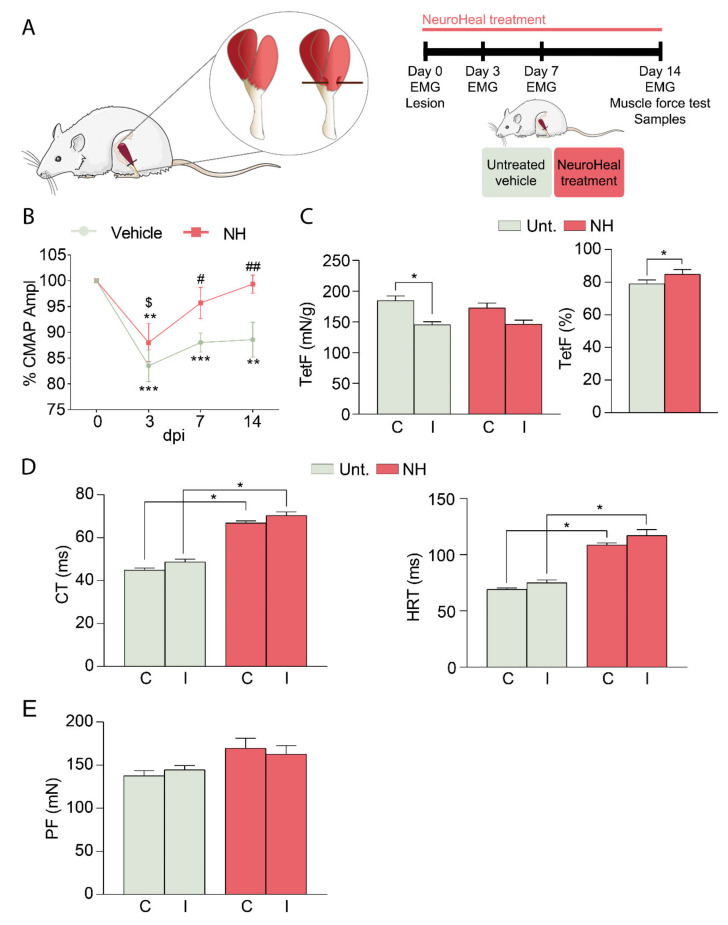
NeuroHeal-treated animals show a better functional recovery after muscle injury. (**A**) Schematic workflow and experimental groups. (**B**) Percentage of the compound muscle action potential (CMAP) amplitude of the ipsilateral gastrocnemius (GA) muscle with respect to the contralateral one from injured untreated (Unt., *n* = 13) and injured treated with NeuroHeal (NH, *n* = 11) groups (two-way ANOVA, * *p* < 0.05, ** *p* < 0.01, *** *p* < 0.001 vs. 0 dpi for each respective group; # *p* < 0.05, ## *p* < 0.01 vs. Unt. for each dpi; $ *p* < 0.05 vs. 3 dpi in NH group). (**C**) Left, bar graph of the average of the maximum tetanus force (TetF) from ipsi- (I) and contralateral (C) GA muscle from Unt. and NH at 14 dpi (*n* = 5; one-way ANOVA, * *p* < 0.01). Right, bar graph of the percentage of TetF of the ipsilateral GA muscle respect to the contralateral one from Unt. and NH at 14 dpi (*n* = 5; *t*-test * *p* = 0.05). (**D**) Left, bar graph of the average of the contraction time (CT) from ipsi- (I) and contralateral (C) GA muscle from GA from Unt. and NH at 14 dpi (*n* = 5; one-way ANOVA, * *p* < 0.0001). Right, bar graph of the average of the half-relaxion time (HRT) from ipsi- (I) and contralateral (C) GA muscle from Unt. and NH at 14 dpi (*n* = 5; one-way ANOVA, * *p* < 0.0001). (**E**) Bar graph of the average of the peak force (PF) from ipsi- (I) and contralateral (C) GA muscle from GA from Unt. and NH at 14 dpi (*n* = 5; one-way ANOVA).

The lesion in the vehicle group caused a reduction of the CMAP amplitude in the GA muscle (83.5% ± 3.1%) compared to the contralateral non-injured muscle at 3 dpi (Figure 1B). Weekly follow-up of the injured animals showed a progressive recovery of the CMAP amplitude until normal values were reached at 14 dpi in the NeuroHeal group, in comparison to the persistent reduction of the CMAP amplitude in the GA at 14 dpi in the untreated group. These results evidenced that the treatment with NeuroHeal significantly improved muscle fiber recovery.

No significant differences were detected in GA muscle weight between the two groups and between ipsi- and contralateral GA muscle for each group at the end of follow-up (Figure S1). Regarding muscle force recovery, untreated animals showed an approximate 20% decrease in TetF of the injured versus contralateral leg (79.1% ± 2.3%), in comparison to NeuroHeal-treated group (84.9% ± 2.9%), without significant differences (Figure 1C). Moreover, the untreated group showed a shorter CT and HRT in comparison to the NeuroHeal-treated group (Figure 1D), coinciding with a PF non-significant increase in this group (Figure 1E). All these data suggested that NeuroHeal may be altering the muscle contractile properties regardless of injury.

#### 3.1.2. NeuroHeal Modulates Fiber Regeneration and Reduces Collagen Deposition

**Figure 2:**
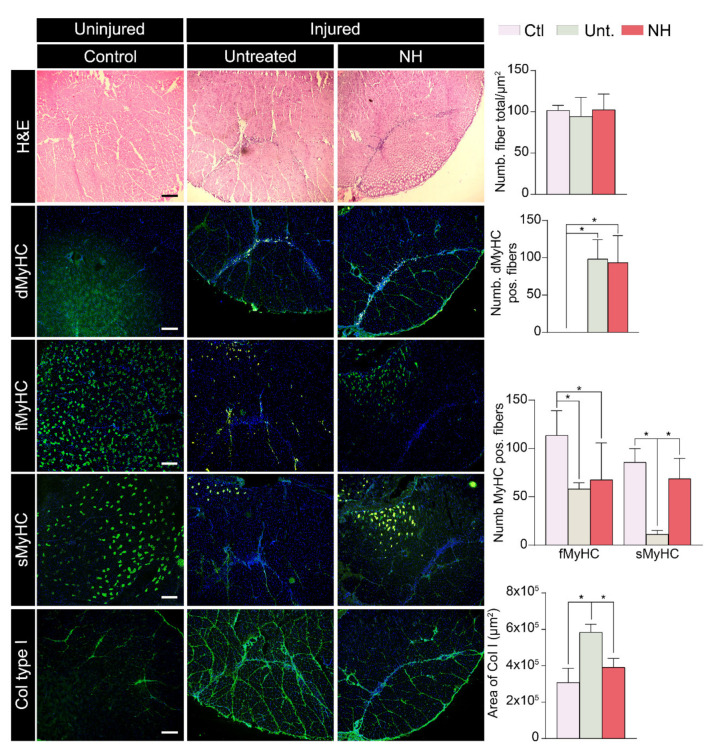
NeuroHeal reduces collagen deposition and promotes slow-twitch fiber typing after muscle injury. ***Left***, representative microphotographs of contralateral control healthy gastrocnemius (GA) muscle sections (Ctl) and lesioned GA muscle sections from the injured untreated (Unt.) and injured treated with NeuroHeal (NH) groups at 14 dpi. Scale bar 500 μm, for all corresponding microphotographs as represented in the first image panel, the control condition. ***First panel***, *left*, representative microphotographs of GA muscle sections stained with H&E. *Right*, bar graph of the average number of muscle fibers (*n* = 4; *t*-test). ***Second panel***, *left*, representative microphotographs of GA muscle sections immunostained for developmental MyHC. *Right*, bar graph of the average number of positive fibers for dMyHC related to the total area of the image (*n* = 4; *t*-test). ***Third and fourth panels***, *left*, representative microphotographs of GA muscle sections immunostained for fast and slow MyHC. *Right*, bar graph of the average number of positive fibers for fMyHC and sMyHC related to the total area of the image (*n* = 3; *t*-test, * *p* < 0.01). ***Fifth panel***, *left*, representative microphotographs of GA muscle sections immunostained for Collagen type I (Col I). *Right*, bar graph of the average of the area immunolabeled for Col I (*n* = 4; *t*-test, * *p* < 0.05).

To determine the effect of NeuroHeal of muscle repair, we analyzed the lesion area, previously identified by H&E staining, with immunohistochemical labeling of markers of regenerating myofibers and of collagen deposition at 14 dpi. The number of total muscle fibers was not modified by the treatment (Figure 2 and Figure S2). Then, we evaluated the regeneration state of the injured fibers. We observed that NeuroHeal did not promote a change in the number of developmental MyHC (Figure 2 and Figure S2). In addition, we observed that NeuroHeal induced a non-significant increase in the number of fast MyHC fibers, and an increase of slow MyHC fibers (Figure 2 and Figure S2) in the injured GA muscle, at the lesion zone. On the other side, NeuroHeal treatment did not modify the distribution of fast and slow MyHC in the contralateral GA muscle (Figure S3). Finally, the NeuroHeal-treated group showed lower collagen I levels in comparison to the untreated group (Figure 2 and Figure S2).

#### 3.1.3. NeuroHeal Influences Muscle Satellite Cells

**Figure 3:**
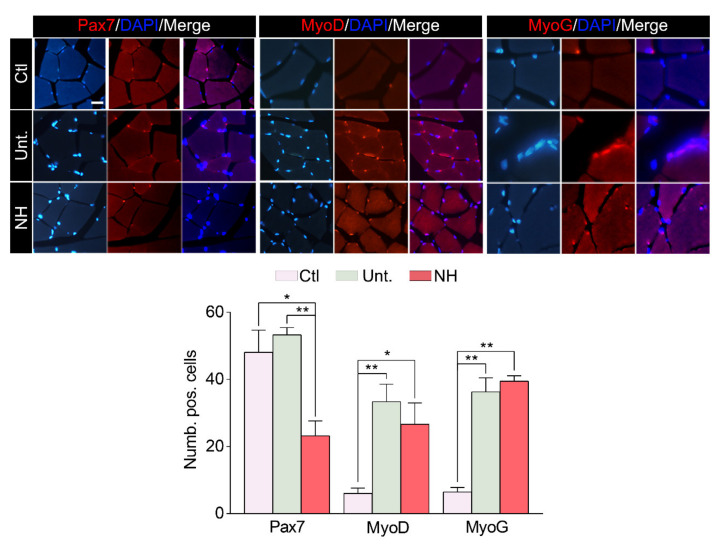
NeuroHeal accelerates satellite cell activation and differentiation after muscle injury. Top, representative microphotographs of the ipsi- and contralateral lesioned gastrocnemius muscle sections revealing the presence of Pax 7, MyoD, and MyoG and stained with DAPI (blue) from the different experimental groups: control (Ctl), injured untreated (Unt.), injured treated with NeuroHeal (NH) at 14 dpi. Scale bar 200 μm and identical for all corresponding microphotographs as represented in the first image panel, the control condition. Bottom, bar graphs of the average number of positive satellite nuclei for Pax7, MyoD, or Myogenin (*n* = 3; one-way ANOVA, * *p* < 0.05, ** *p* < 0.01).

We further investigated if NeuroHeal might influence the response of SCs, which are the natural/endogenous way of muscle to recover after injury by regeneration, as they are able to differentiate into myofibers [21]. At 14 dpi, the number of Pax7-positive cells (a classical marker of activated SCs) was similar in the untreated injured animals compared to uninjured controls, while NeuroHeal treatment significantly reduced the quantity (Figure 3). In contrast, there was a significant increase in the number of MyoD- (myoblast marker) and MyoG-positive cells (Myogenin, myocyte marker) in injured animals regardless of the treatment, as expected, which suggests commitment of SCs in the myogenic process (Figure 3).

**Figure 4:**
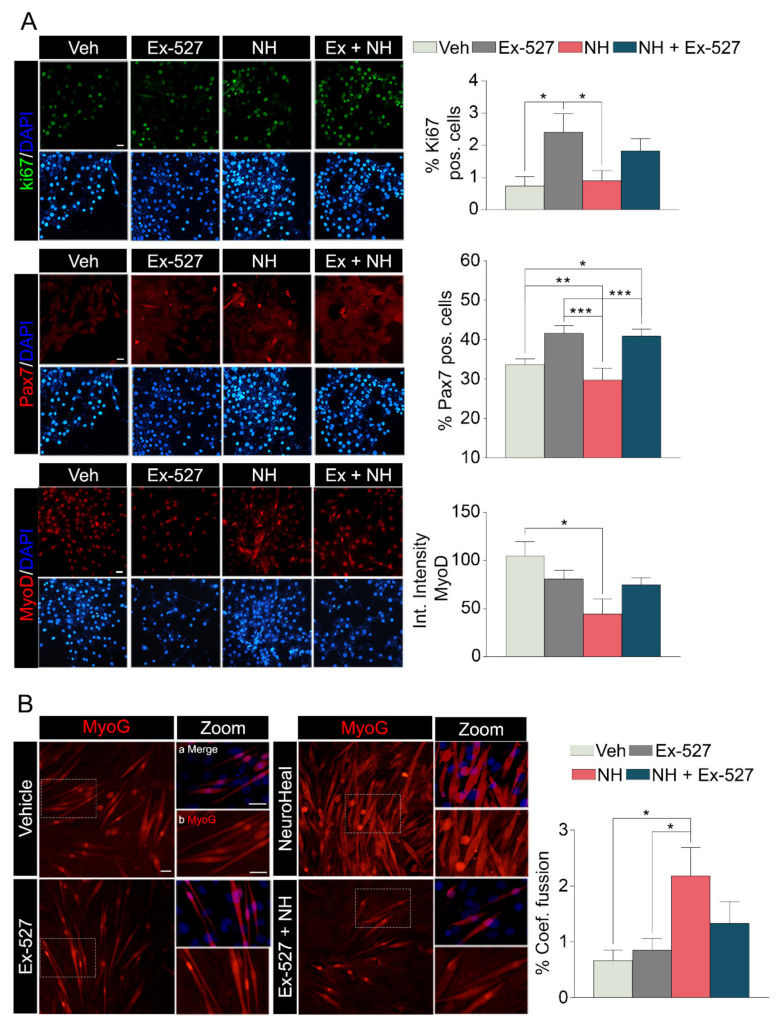
NeuroHeal modulates differentiation process and increases myoblast fusion in vitro. (**A**) Left, representative microphotographs of differentiated C2C12 myoblast cell line immunostained for Ki67, Pax7, and MyoD and stained with DAPI from the different experimental groups at 3 days of differentiation: atrophy-induced treated with vehicle (Veh), atrophy-induced treated with Ex-527 (Ex-527), atrophy-induced treated with NeuroHeal (NH), and atrophy-induced treated with NH plus Ex-527. Scale bar 25 μm and identical for all corresponding microphotographs as represented in the first image panel, the vehicle condition. Right, bar graph of the average number of positive nuclei for Ki67 and Pax7, and the relative intensity per cell of MyoD (*n* = 3; one-way ANOVA, * *p* < 0.05, ** *p* < 0.01, *** *p* < 0.001). (**B**) Left, representative immunofluorescence microphotographs of differentiated C2C12 myoblast cell line immunostained for MyHC and stained with DAPI (blue) from the different experimental groups at 5 days of differentiation. Panels a and b are zoom images for each condition from the images of the left. The scale bar is 200 μm for all corresponding microphotographs as represented in the first image panel, the vehicle condition, and zoomed images. Right, bar graph of the percentage of the number of positive myotubes with three or more nuclei (*n* = 3, one-way ANOVA, * *p* < 0.05).

We additionally analyzed the differentiation process of myoblasts in vitro to verify the implication of NeuroHeal in this process. The differentiation process of the myoblast cell line C2C12 occurs during 3–5 days of low-serum culture medium [22]. The non-activated and non-quiescent SCs are positive for Ki-67 and negative for Pax7. The following day after the initiation of the differentiation process, the number of Ki-67 positive cells was similar to the non-differentiated cells, but it drastically dropped after 3 div culture (Figure S4B). The use of Ex-527, a selective inhibitor of SIRT1, increased the number of Ki-67 positive and Pax7 positive cells, while the total number of cells was similar to the control (Figure S4C), suggesting the arresting of the differentiation process at this stage in a proportion of cells (Figure 4A). The relative intensity of MyoD within the cells treated with Ex-527 was similar to the control in agreement with normal low activity of SIRT1 at this stage. Treatment with NeuroHeal did not affect the number of Ki-67 positive cells but significantly diminished the number of Pax7 positive cells with respect to the control, suggesting that more cells entered into later stages of differentiation (Figure 4A). Curiously, the number of MyoD positive cells was not affected by NeuroHeal treatment despite the lower intensity of its expression compared to the control (Figure 4A). This observation is consistent with the fact that SIRT1 activation reduces the expression of MyoD [23]. However, the coefficient of fusion of the formed mature myotubes was increased with NeuroHeal compared to the untreated condition, suggesting an acceleration of this differentiation process (Figure 4B). All these effects produced by NeuroHeal were not observed if Ex-527 was concomitantly added to the wells, suggesting that the activation of SIRT1 might be involved, although other factors may play a role and compensating some effects. Finally, we analyzed parvalbumin levels in vivo and in vitro to verify the interaction between SIRT1 and parvalbumin in the differentiation process [24]. NeuroHeal treatment showed a reduction in the relative intensity of parvalbumin on myotubes of C2C12 cells (Figure S5A) and also in the in vivo analysis in the intact contralateral GA muscle (Figure S4B). This reduction was reverted by the addition of Ex-527 (Figure S5A). Altogether, these results suggest that NeuroHeal modulates the differentiation process of the myoblast C2C12 cell line in vitro, increasing the fusion of the new formed matured myotubes, and that SIRT1 activation is involved on it.

## 4. Discussion

The results of this work indicate that NeuroHeal affects muscle cell biology, improving muscle regeneration and accelerating myogenesis after injury. These beneficial effects are accompanied by reduced collagen deposition and an increased number of mature muscle fibers, preferentially of the slow-twitch type. Overall, NeuroHeal may be a promising treatment to improve skeletal muscle regeneration after damage and for some muscle-related diseases.

Although NeuroHeal was discovered to act on the nervous system, in previous studies, we observed that it also reduces muscle atrophy caused by denervation [12] and by hindlimb immobilization [17]. For that reason, we pursued to decipher whether it may act on muscle cell biology as well by using a rat model of sports-type muscle injury [20]. Surprisingly, we observed that the treatment with NeuroHeal advanced the recovery of the electrophysiological response, and promoted a tendency to increase the TetF of the damaged muscle. This was accompanied by observations in the histological analysis; less collagen deposition, which contributes to form scar tissue, and an increasing number of mature muscle fibers, in particular slow-twitch fibers. These observations are in agreement with the known mechanism of action of NeuroHeal as a SIRT1 activator [18,25]. Several studies have shown that activation of SIRT1 in muscle disease models promotes shifting from fast to slow-twitch fibers [26,27,28]. However, we did not observe such an increase in this type of fibers in the uninjured muscles in our model, suggesting that more time of treatment is necessary under normal conditions or that this only occurs when the muscle is affected/lesioned. In any case, it would be worth exploring these possibilities.

In the muscular force analysis, there was an unexpected increase in the contraction and relaxation time produced by NeuroHeal treatment, due to a PF light increase. This may be attributed to an increase in the pulse of free Ca^+2^ in the cytoplasm. Two possible mechanisms may contribute to this action. First, it is reported that one action of NeuroHeal’s compound ACA is the activation of the VGCC channel [29], which provides Ca^+2^ entry activating the RyR. This prolongation of the RyR activation could explain the increase in the CT parameter. A second reason may be related to the reduction of parvalbumin produced by NeuroHeal, which acts as a Ca^+2^ binding protein, extending the concentration of free Ca^+2^. This would impede the muscle to relax, leading to an HRT increase. In addition, these low levels are in agreement with findings reported by Ducreux and collaborators [24]. They reported that parvalbumin expression and mitochondrial volume in muscle cells are inversely regulated via a SIRT1/PGC-1α signaling axis. We did not explore the action of NeuroHeal on mitochondria biogenesis, but considering the importance of parvalbumin in buffering Ca^+2^ during the relaxation/contraction process, this may explain why the times for contraction and relaxation were higher in the animals treated with NeuroHeal compared to controls in both contralateral and ipsilateral muscles.

Finally, we explored the effects of NeuroHeal on SCs, which normally remain in a quiescent state until they receive activating signals to begin proliferation [30,31]. SC activation is metabolically highly demanding, and it is associated with a large increase in cellular ATP. Indeed, it was found that SIRT1, a key nutrient sensor, modulates autophagic flux during SC activation [32]. The activation of the autophagic machinery by SIRT1 is necessary in order to generate nutrients, which are essential for the generation of ATP, to support the considerable increase in anabolic activity associated with the activation process. Inhibition of SIRT1 or autophagy causes a delay in SC activation [33]. We have previously found that NeuroHeal activates SIRT1 and favors the autophagy flux [14], so these may be causing either the effect of a quick activation of myoblast observed in vitro or the diminished number of Pax7 positive SC in vivo compared to the untreated animals. The same number of Pax7 positive SC in vivo in the untreated and control groups could be caused by a delayed activation of the SC in contrast to an early induction due to SIRT1 activation. Upon SC activation, the metabolic switch from fatty acid oxidation to glycolysis produces a drop in free NAD+, and SIRT1 loses its catalytic activity, which allows the expression of muscle-specific genes, such as MyoD [34]. We observed that NeuroHeal treatment did not prevent the ability of cells to finish myogenesis and even reduced MyoD expression, which is in agreement with what is reported on SIRT1 action [34]. Furthermore, we observed an increase in the fusion coefficient to form myotubes with NeuroHeal treatment, which, again, could be related to the activation of SIRT1 and its favorable action in the respiration of mitochondria [35].

Overall, we conclude that NeuroHeal could be used clinically to accelerate muscle regeneration, and probably also as a treatment in various muscle diseases due to its ability to activate SIRT1 [36].

## Data Availability

The data presented in this study are available on request from the corresponding author. The data are not publicly available due to patent issues.

## Supplementary Materials

The following are available online at https://www.mdpi.com/2073-4409/10/1/22/s1, Figure S1: Muscle injury does not modify muscle weight. Figure S2: NeuroHeal reduces collagen deposition and promotes slow-twitch fiber typing after muscle injury. Figure S3: NeuroHeal does not modify the myosin-fiber pattern in healthy muscle. Figure S4: C2C12 cell line maintains a high rate of proliferation one day after differentiation. Figure S5: Parvalbumin is modulated by SIRT1 in vivo and in vitro.

## Special Statement

The authors wish to dedicate a special mention in memory of Caty Casas, an influential scientist, and a wonderful and brave person, who died prematurely at the age of 52 years. It has been an honor and a privilege to share part of her life, her professional capacity, and her ethical and human values.
