# Supplementary material for: NeuroHeal Improves Muscle Regeneration after Injury
Source: Cells. 2020 Dec 24;10(1):22. doi: 10.3390/cells10010022 (PMC7824727; doi:10.3390/cells10010022)
Supplement: Supplementary file 1 [file cells-10-00022-s001.pdf]

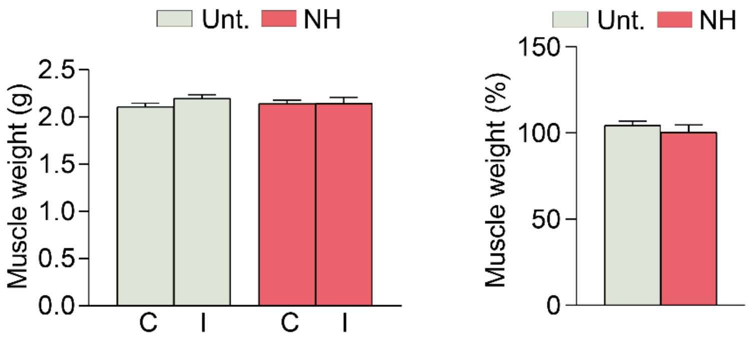

Figure S1. Muscle injury does not modify muscle weigh**t.** Left, bar graph of the average of the muscle weight from ipsi- and contralateral gastrocnemius (GA) muscle from the different experimental groups injured untreated (Unt.) and injured treated with NeuroHeal (NH) at 14 dpi (n = 5; t-test). Right, bar graph of the relative mean of muscle weight of the ipsilateral GA muscle respect to the contralateral one from Unt. and NH at 14 dpi (n = 5; t-test).

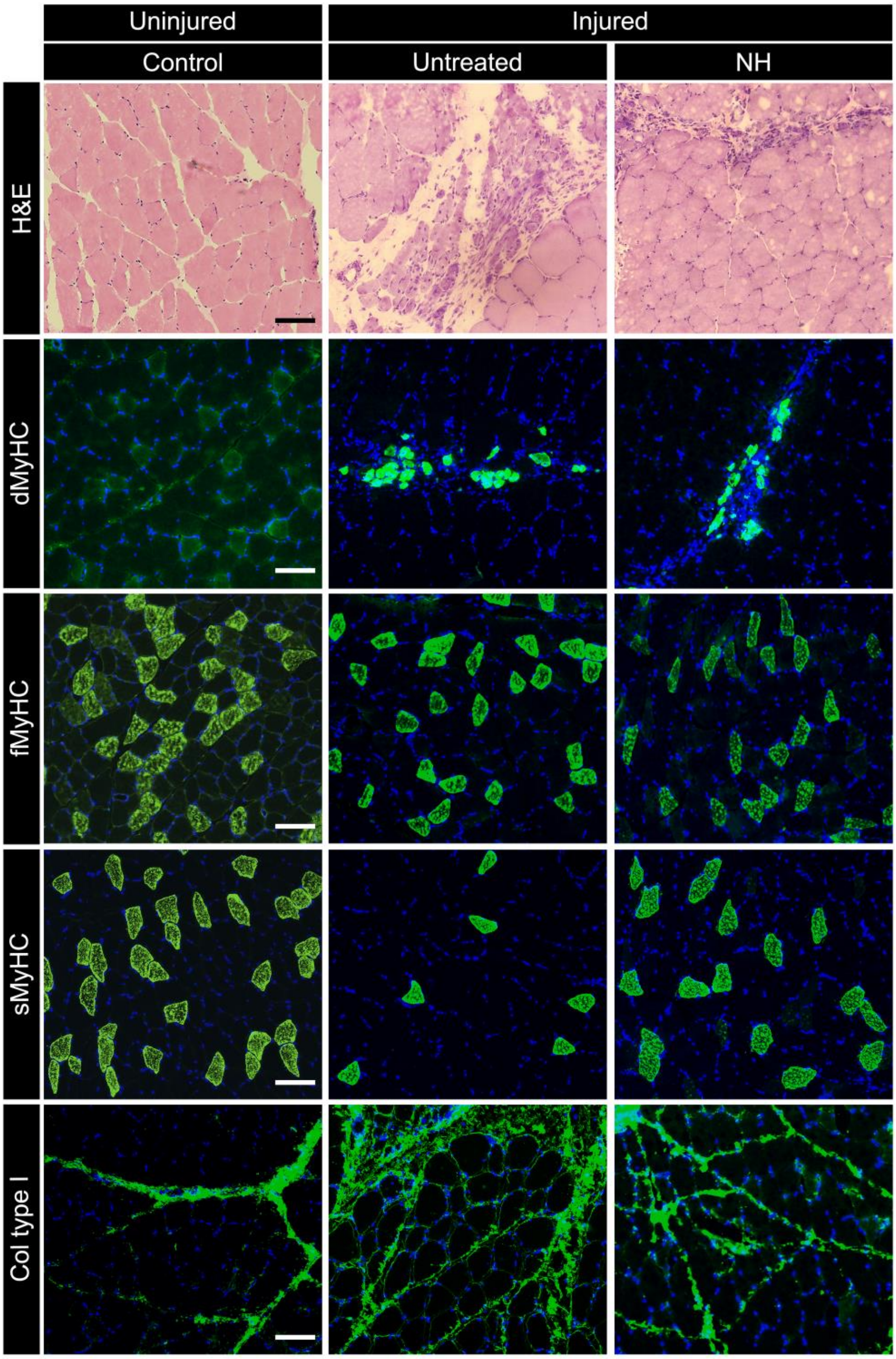

Figure S2. NeuroHeal reduces collagen deposition and promotes slow-twitch fiber typing after **muscle injury.** Zoom images of contralateral control healthy gastrocnemius (GA) muscle sections (Ctl) and lesioned GA muscle sections from the injured untreated (Unt.) and injured treated with NeuroHeal (NH) groups at 14 dpi. Scale bar 100 µm, for all corresponding microphotographs as represented in the first image panel, the control condition. First panel, representative microphotographs of GA muscle sections stained with H&E. Second panel, representative microphotographs of GA muscle sections immunostained for developmental MyHC. Third and fourth panels, representative microphotographs of GA muscle sections immunostained for fast and slow MyHC. Fifth panel, representative microphotographs of GA muscle sections immunostained for Collagen type I (Col I).

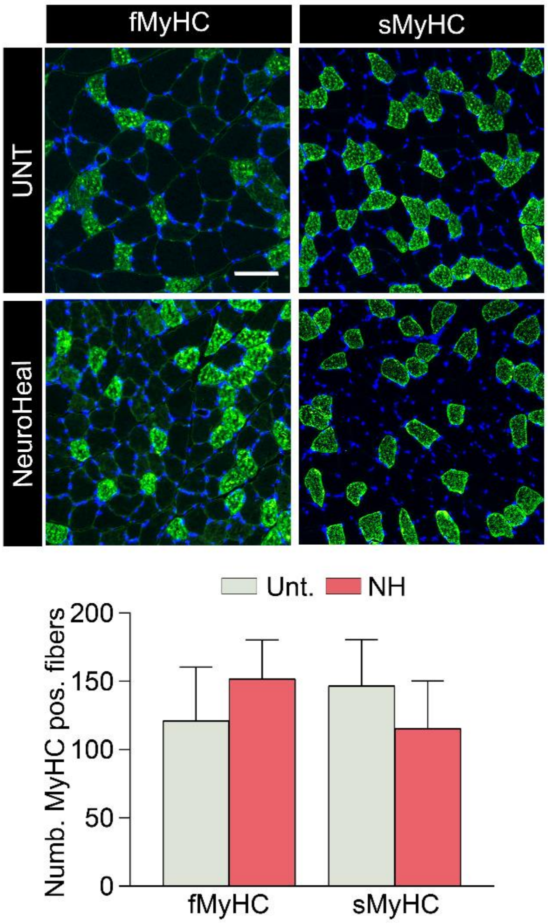

Figure S3. NeuroHeal does not modify the myosin-fiber pattern in healthy muscle. Top, representative microphotographs of the contralateral lesioned gastrocnemius muscle sections revealing the presence of fast and slow MyHC with DAPI (blue) from the different experimental groups injured untreated (Unt.) and injured treated with NeuroHeal (NH) at 14 dpi. Scale bar 100 μm and identical for all corresponding microphotographs as represented in the first image panel, the untreated condition. Bottom, bar graphs of the average of positive fibers for fast and slow MyHC (n = 5; t-test).

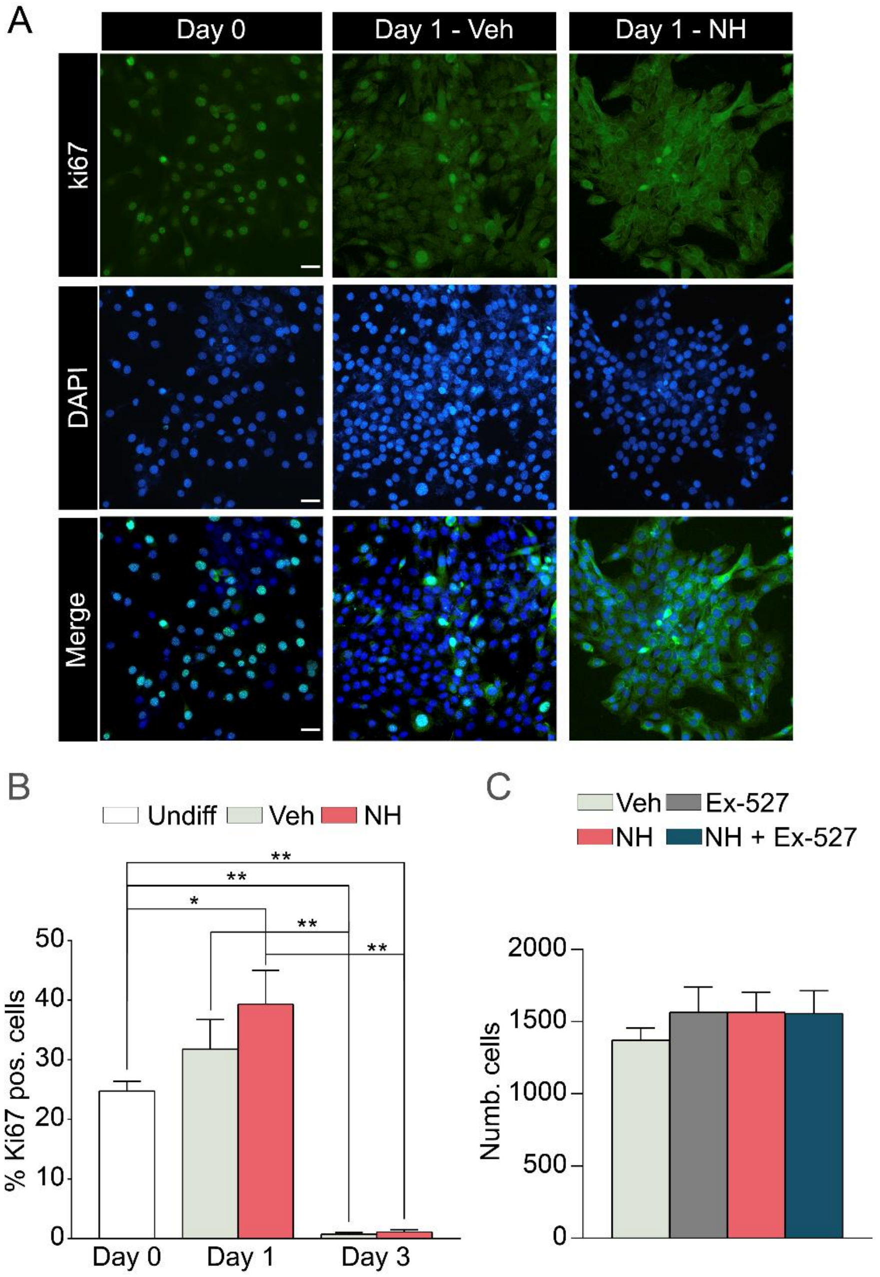

Figure S4. C2C12 cell line maintains high rate of proliferation 1 day after differentiation. (a) Representative microphotographs of differentiated C2C12 myoblast cell line immunostained for Ki67 and stained with DAPI from the different experimental groups at day 0 and 1 days of differentiation: atrophy-induced treated with vehicle (Veh) and atrophy-induced treated with NeuroHeal (NH). Scale bar 50 µm and identical for all corresponding microphotographs as represented in the first image panel, the day 0. (b) Bar graph of the average number of positive nuclei for Ki67 (n = 3; one-way ANOVA, * $p < 0.0001$). (**c**) Bar graph of the average number of nuclei for condition (n = 3; one-way ANOVA).

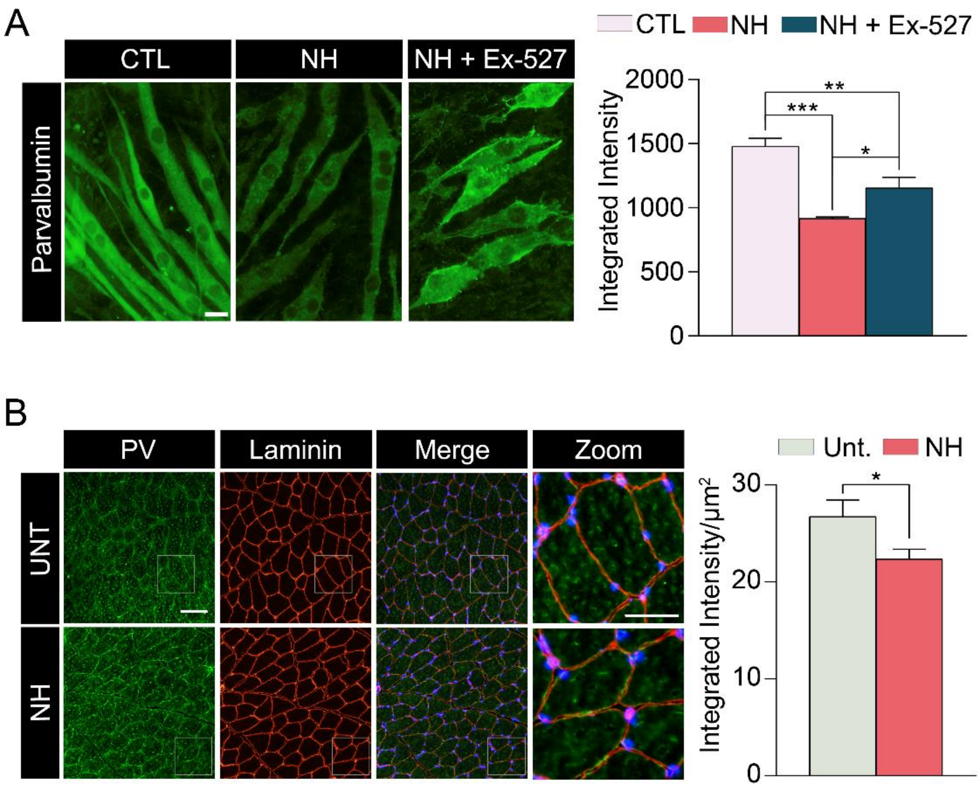

Figure S5. Parvalbumin is modulated by SIRT1 in vivo and in vitro. (a) Left, representative microphotographs of differentiated C2C12 myoblast cell line immunostained for parvalbumin and stained with DAPI from the different experimental groups at 3 days of differentiation: untreated (Ctl), treated with NeuroHeal (NH) and treated with NH plus Ex-527. Scale bar 25 µm and identical for all corresponding microphotographs as represented in the first image panel, the control. Right, bar graph of the relative intensity per cell of parvalbumin (n = 5; one-way ANOVA, * $p < 0.05$, ** $p < 0.1$, *** $p < 0.001$). (b) Left, representative microphotographs of the contralateral lesioned gastrocnemius muscle sections revealing the presence of parvalbumin, laminin with DAPI (blue) from the different experimental groups injured untreated (Unt.) and injured treated with NeuroHeal (NH) at 14 dpi. Scale bar 100 µm and identical for all corresponding microphotographs as represented in the first image panel, the untreated condition. Right, bar graphs of the relative intensity per fiber of parvalbumin (n = 5, t-test, * $p < 0.05$).
